# Antibacterial and sunlight-driven photocatalytic activity of graphene oxide conjugated CeO_2_ nanoparticles

**DOI:** 10.1038/s41598-024-54905-0

**Published:** 2024-03-19

**Authors:** Fauzia, Mo Ahamad Khan, Mohd Chaman, Ameer Azam

**Affiliations:** 1 Department of Applied Physics, Z.H. College of Engineering & Technology, Aligarh Muslim University, Aligarh, 202002 India; 2 Department of Physics, Faculty of Science, Islamic University of Madinah, 42351 Madinah, Saudi Arabia; 3 Department of Microbiology, Jawaharlal Nehru Medical College, Aligarh Muslim University, Aligarh, UP 202002 India; 4 Mewat Engineering College, Nuh, Mewat, Haryana 122107 India

**Keywords:** Graphene oxide, Cerium oxide, Sonication, Nanocomposite, Photocatalytic activity, Antibacterial activity, Nanoscience and technology, Physics

## Abstract

This work focuses on the structural, morphological, optical, photocatalytic, antibacterial properties of pure CeO_2_ nanoparticles (NPs) and graphene oxide (GO) based CeO_2_ nanocomposites (GO-1/CeO_2_, GO-5/CeO_2_, GO-10/CeO_2_, GO-15/CeO_2_), synthesized using the sol–gel auto-combustion and subsequent sonication method, respectively. The single-phase cubic structure of CeO_2_ NPs was confirmed by Rietveld refined XRD, HRTEM, FTIR and Raman spectroscopy. The average crystallite size was calculated using Debye Scherrer formula and found to increase from 20 to 25 nm for CeO_2_ to GO-15/CeO_2_ samples, respectively. The related functional groups were observed from Fourier transform infrared (FTIR) spectroscopy, consistent with the outcomes of Raman spectroscopy. The optical band gap of each sample was calculated by using a Tauc plot, which was observed to decrease from 2.8 to 1.68 eV. The valence state of Ce (Ce^3+^ and Ce^4+^) was verified using X-ray photoelectron spectroscopy (XPS) for CeO_2_ and GO-10/CeO_2_. The poisonous methylene blue (MB) dye was used to evaluate the photocatalytic activity of each sample in direct sunlight. The GO-15/CeO_2_ nanocomposite showed the highest photocatalytic activity with rate constant (0.01633 min^–1^), and it degraded the MB dye molecules by 100% within 120 min. The high photocatalytic activity of this material for degrading MB dye establishes it as an outstanding candidate for wastewater treatment. Further, these nanocomposites also demonstrated excellent antimicrobial activity against *Pseudomonas aeruginosa* PAO1.

## Introduction

Water pollution is a big concern for scientists, environmentalists, and company owners worldwide. This is because wastewater from the textile, food, leather, and chemical sectors releases toxic substances into the environment^1,2^. These soluble hazardous chemicals have a negative impact on aquatic life, and many of these molecules of chemicals are resistant to destruction by light, acids, bases, and oxygen. Thus, they persist in the environment at large. These dyes and other chemicals are removed from the water using a variety of techniques such as adsorption^3,4^, photocatalysis^4–8^, ion exchange^9,10^, coagulation, chemical oxidation^11,12^, etc. In recent years, photocatalysis has gained popularity as an approach to dye degradation because it yields carbon dioxide and water as byproducts and prevents secondary water contamination. This method is not only environmentally friendly but also simple and economical^13–16^.

Furthermore, the increased persistence of chronic infections, especially resistance to the bacteria *Pseudomonas aeruginosa*, makes bacterial infections one of the key global public health concerns. *Pseudomonas aeruginosa* is a common cause of conciliatory and hospital-attained infections^17^. *P. aeruginosa* infection may cause severe consequences in immunocompromised individuals and those with burn infections, respiratory, and urinary tract infection, cystic fibrosis, sepsis, osteomyelitis, and endocarditis^18,19^. Antibiotic overuse has led to the transmission of multidrug-resistant (MDR) *P. aeruginosa* infections^20^. One of the most important virulence factors for *P. aeruginosa* is its ability to build a biofilm. Biofilm-forming cells are more resistant to the host immune system and antibiotics^21^. The process of creating novel antibiotics is expensive and time-consuming. Furthermore, quick advances in resistance shorten the shelf life of antibiotics^22,23^. New therapeutic approaches are therefore required to address the problem of MDR organisms.

Various photocatalysts and antibacterial agents, including CdS^24^, TiO_2_^25^, g-C_3_N_4_^26^, ZnO^27^, etc., have been studied in cleaning the environment and biomedical applications. Some of these metal oxide semiconductors, including CeO_2_, α-Fe_2_O_3_^28^, TiO_2_^25^, and ZnO^27^, exhibit high photocatalytic activity because of their exceptional qualities, which include chemical stability and ease of production. The rare earth oxide ceria (CeO_2_), one of the most well-liked metal oxide semiconductors, is extensively used in a variety of industries, including oxygen sensors^29^, fuel cells^30^, and solar cells^31^. CeO_2_ NPs have high oxygen storage capacity and plentiful oxygen vacancies (V_o_), strong catalytic properties due to the reversible Ce^3+^/Ce^4+^ pairs, and resistance to photo-corrosion, as reported in earlier studies^32,33^. A study highlighted the exceptional photocatalytic activity of CeO_2_ NPs in the degradation of MB dye under visible light irradiation. Their findings not only demonstrated the efficacy of CeO_2_ NPs but also emphasized the need for further investigation to elucidate the mechanisms and optimize the photocatalytic process^34^. Although this CeO_2_ (n-type semiconductor) has a broad bandgap of 2.8–3.1 eV, it can absorb light only up to 400 nm in the visible and UV spectra, which account for less than 5% of the energy in the solar spectrum^35^. CeO_2_ has certain limitations to use as a photocatalyst due to its wide bandgap, some techniques, such as noble metal deposition, doping, surface photosensitization, and solid-solution creation, can be used to decrease its bandgap in order to utilize it for a broad spectrum of sunlight.

Doping graphene oxide (GO) into cerium oxide (CeO_2_) offers significant advantages that enhance the performance of this composite material in various applications. GO, with its two-dimensional structure and exceptional electrical conductivity, acts as an ideal dopant for CeO_2_^36,37^. Firstly, GO doping effectively narrows the wide bandgap of CeO_2_, extending its light absorption capacity into the visible spectrum, which is crucial for photocatalytic applications. This enhanced light absorption capability allows for more efficient utilization of solar energy and results in improved photocatalytic activity. Additionally, the incorporation of GO enhances the charge separation and transport properties within the CeO_2_ matrix, reducing the recombination of photoinduced electron–hole pairs. This leads to higher photocatalytic efficiency and the generation of more reactive oxygen species for pollutant degradation.

As a result, the present research produces the photocatalysts that can be used in air purification^38^, water splitting^39^, organic pollutants degradation, and CO_2_ conversion^40^. There have been limited studies of GO-based CeO_2_ materials working simultaneous as photocatalysts and antibacterial agents. Here, GO-based CeO_2_ nanocomposites can act simultaneously as a good photocatalyst as well as efficient antibacterial agent. The photocatalytic and antibacterial activities of every sample were investigated against methylene blue (MB) dye and *Pseudomonas aeruginosa*, respectively. The unique properties of Methylene Blue, including its solubility, stability, color, and responsiveness to light, make it a versatile and commonly used dye in wastewater treatment. Its inclusion in studies allows for a better understanding of photocatalytic processes and the development of effective water treatment technologies.

In the current research work, CeO_2_ and GO-based CeO_2_ photocatalysts for the degradation of toxic dyes are synthesized using sol–gel auto-combustion and sonication methods, respectively. Cerium nitrate and citric acid were mixed in water, with citric acid gradually added to the cerium nitrate solution at room temperature. Ethylene glycol was introduced to boost the reaction rate and ammonia solution was used to maintain pH to 7. The temperature was raised to 120 °C to create a viscous gel. The gel was heated to 200 °C to obtain burnt powder and calcined at 500 °C for 3 h, resulting in the formation of CeO_2_ nanoparticles. Synthesis of CeO_2_ NPs using this method is unique and cost-effective, as reported by different researchers so far. This work is aimed to enhance the photocatalytic activity of CeO_2_ by incorporation of GO and also to explore their antibacterial activity. These photocatalysts are highly activated in the visible light. The obtained result shows that the photocatalytic and antibacterial activity are very high for GO-15/CeO_2_ nanocomposite in comparison to other synthesized materials.

## Materials and methods

### Materials

Cerium nitrate (Ce(NO_3_)_3_.H_2_O) with 99.99% purity and graphite powder, hydrogen peroxide (H_2_O_2_:30%), and ethanol (C_2_H_2_OH) were purchased from CDH Pvt. Ltd., New Delhi, India. Potassium permanganate (KMnO_4_) and citric acid (C_6_H_8_O_7_.H_2_O) were purchased from RFCL (Rankem) Ltd., New Delhi, India. Ethylene glycol (CH_2_OH.CH_2_OH), hydrochloric acid (HCl: 37%), and sulphuric acid (H_2_SO_4_) were purchased from Thermo Fisher Scientific Pvt. Ltd., Mumbai, India. All chemicals were used without any further purification.

### Synthesis of cerium oxide NPs

CeO_2_ was synthesized using a low-cost auto-combustion method. Firstly, cerium nitrate and citric acid were added to 80 ml and 20 ml of distilled water separately at room temperature. A gradual addition of citric acid solution to the cerium nitrate solution was done with constant stirring. To enhance the reaction rate, 2 ml ethylene glycol was added after stirring for half an hour, and the pH was adjusted to 7 using ammonia solution during stirring, subsequently the temperature was increased to 120 °C to get the viscous gel. The burnt powder, obtained after increasing the temperature to 200 °C, was calcined at 500 °C for 3 h to obtain the CeO_2_ NPs.

### Synthesis of graphene oxide (GO) nanosheets

GO was prepared successfully using a modified Hummer’s method. First of all, 2 g of fine graphite powder was added to 150 ml of concentrated sulphuric acid (H_2_SO_4_) with continuous stirring in the ice bath for 30 min. When the temperature reached 5 °C, 8 g of KMnO_4_ was added very slowly to this mixture of graphite powder and H_2_SO_4_ to obtain the green solution. After one hour of stirring, the sample was removed from the ice bath, and the temperature was kept constant at 35 °C with continuous stirring for 24 h. Further, 200 ml distilled water was added to dilute the mixture and left to stir for 15 min. In order to quench the reaction, 30 ml H_2_O_2_ (30%) was added to the solution, stirred for 1 h, and left overnight to get precipitate. This final product was centrifuged with 200 ml HCl (10%) and H_2_O_2_ (1%) solution in order to remove impurities, also with distilled water and ethanol to maintain pH ~ 7. Finally, the product was dried in a vacuum oven at 70 °C for 24 h and pulverized into a fine powder form.

### Synthesis of GO-based CeO_2_ nanocomposite

Graphene oxide (GO) based CeO_2_ nanocomposites were systematically synthesized through a controlled sonication method. Precise amounts of GO powder, ranging from 1 to 15 wt.%, were incorporated into a 30 ml volume of deionized water and subjected to ultrasonication for 1 h, ensuring a homogenous dispersion. Subsequently, the as-synthesized CeO_2_ powder was carefully introduced into the GO dispersion with gradual addition during stirring at room temperature. The resulting solution underwent an extended stirring period of 3 h to facilitate thorough integration. To conclude the synthesis, the composite solution was carefully dried in an oven at 80 °C for 24 h, promoting the removal of solvents and yielding well-defined GO-based CeO_2_ nanocomposites with tailored graphene oxide concentrations.

### Materials characterization

The structural properties were investigated using Fourier transform infrared (FT-IR) spectroscopy, X-ray diffraction measurements (XRD), transmission electron microscopy (TEM)/high-resolution transmission electron microscopy (HRTEM), Raman spectroscopy and selected area electron diffraction (SAED) patterns. The optical properties of synthesized materials are measured using UV–visible spectroscopy, and their morphological properties are measured using a scanning electron microscope (SEM) along with EDX. Shimadzu LabX XRD-6100 Cu-k_α_ radiation was used to generate the XRD spectra. The Rietveld refinement was carried out using the FULLPROF program to determine the crystal structure parameters of the pure CeO_2_ and to confirm the single phase. Perkin Elmer spectrum-2 was used to perform FT-IR analysis on each sample to study phase purity and the functional groups. In order to further confirm vibrational frequencies obtained from FTIR, the Laser Raman spectrum has also been recorded because they are complementary to each other. To record Raman spectra, a Renishaw inVia Raman microscope with a 532 nm laser source was utilized. For the morphological study, a JEOL JEM-2100 TEM operating at 200 kV was used; the crystal structure was analyzed by means of the SAED ring network and HRTEM fringe patterns. For morphological investigation, scanning electron microscopy (SEM) was also utilized, and an energy dispersive x-ray (EDS) linked to a JEOL JSM-6510LV SEM operating at 50 kV (JEOL Co., Ltd.) was utilized for elemental analysis. The UV–Vis NIR spectrophotometer Shimadzu UV-1601 was used to measure the absorption spectra of each sample. In addition, photocatalytic activities comprising the decomposition of MB dye were studied using UV–vis absorbance spectra.

### Photocatalytic activity

A 10 ppm stock solution of methylene blue (MB) dye was formed for the photocatalysis experiment in sunlight irradiation (Time: between 11:30 a.m. to 2:00 p.m., date: 18-June 2022, Place: Aligarh, India)^41^. As a control, 5 ml of an aqueous dye solution without a photocatalyst was extracted. After preparing a stock solution of MB dye in 100 ml of distilled water, 20 mg of CeO_2_ NPs and their nanocomposites were added to the solution one at a time while agitating at room temperature in the presence of solar light and at various time intervals (0, 15, 30, 45, 60, 75, 90, 105, and 120 min), 5 ml samples were collected in a transparent container (0, 15, 30, 45, 60, 75, 90, 105, and 120 min). The NPs were entirely removed from the solution using centrifugation. As a result, the UV–Vis spectrophotometer was used to measure each sample's UV–Vis absorbance spectra.

### Antibacterial activity

#### Microorganisms

In the current research, *Pseudomonas* *aeruginosa* PAO1 was chosen, which was collected from the Department of Microbiology, Aligarh Muslim University, Aligarh. The bacterial culture was cultivated in nutrient broth and maintained on nutrient agar slants.

#### Well diffusion method

The antibacterial activity of each sample was evaluated using the well-diffusion method against pathogenic bacteria^42^. A disinfected cotton swab was utilized to spread the bacterial strain over the Mueller–Hinton agar plate. A 6-mm well was made by a sterile borer on agar. In the test, 100 µL of nanoparticles (50 μg/mL) were loaded into the wells. After that, the agar plate was incubated for 24 h at 37 °C. After 24 h of incubation, the plates were studied for the presence of a clear inhibition zone. Distilled water (sterile) was used as the negative control, and a blank well, which did not incorporate any solvent or nanomaterials, was also included.

Antibiotic susceptibility testing for *P. aeruginosa* PAO1 was carried out using automated microbiological systems, specifically the VITEK-2 platform. Furthermore, the investigation included *P. aeruginosa* POA1 strains demonstrating resistance to Amikacin, Cefepime, Ciprofloxacin, Colistin, Gentamicin, and Imipenem.

#### Determination of minimum inhibitory concentration (MIC)

Pure and GO-based CeO_2_ nanoparticles that showed a zone of inhibition against bacteria were subjected to MIC testing. With slight modifications, the MIC of nanoparticles was calculated using the broth microdilution method^43^. A 96-well ELISA plate was used to make a two-fold serial dilution of nanoparticles in Muller-Hinton broth for different concentrations. Bacteria were cultivated in various amounts of samples and incubated at 37 °C for 18 h. After incubation, 20 µl of Triphenlytetrazolium chloride (TTC, HiMedia, India) (3 mg/mL) was added to each well and kept at room temperature for 20 min; the pink color indicates bacterial growth. The MIC was determined as the lowest concentration at which no color change was visible, suggesting the presence of no metabolically active cells.

## Results and discussion

### X-ray diffraction

**Figure 1:**
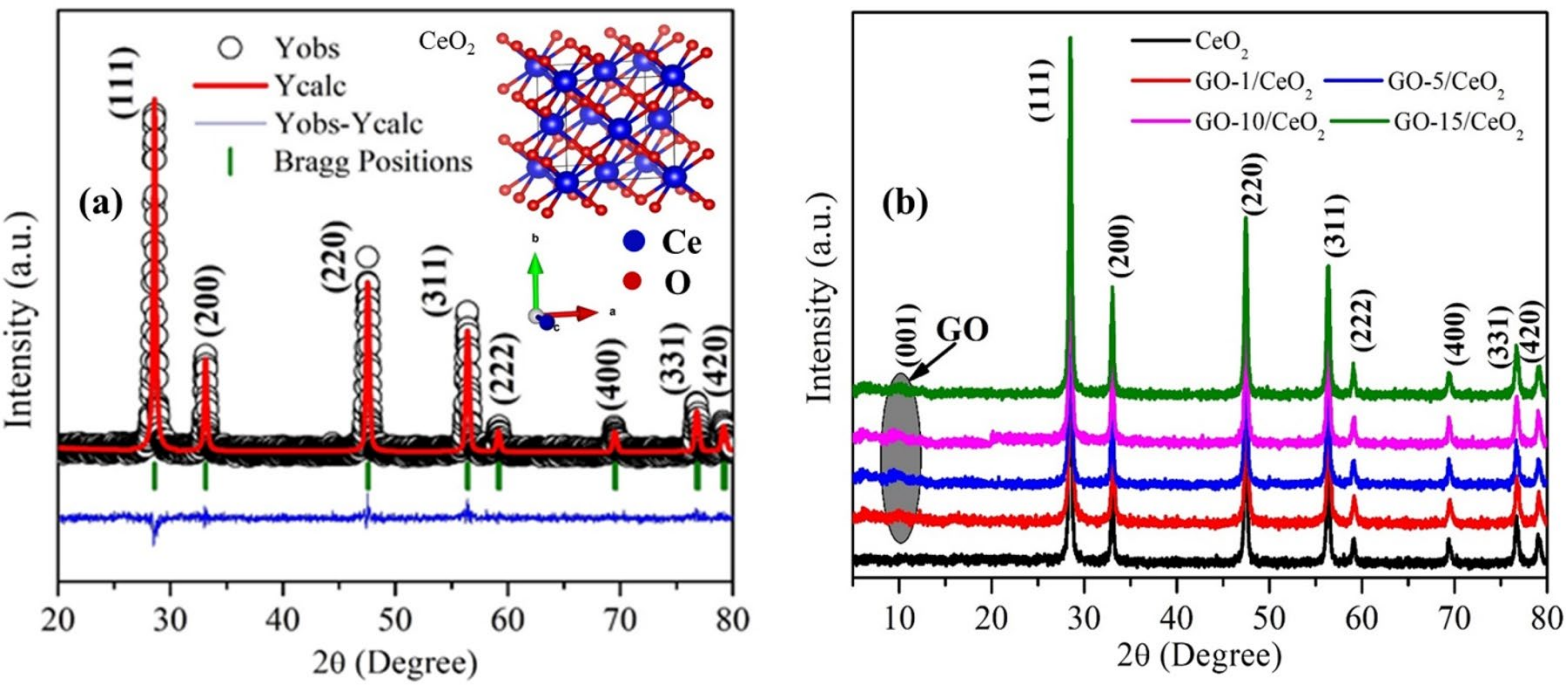
(**a**) Rietveld refined XRD spectrum of CeO_2_ NPs. (**b**) XRD spectra of CeO_2_ NPs and GO (1, 5, 10 and 15 wt %) based CeO_2_ nanocomposite.

XRD spectra of pure CeO_2_ and GO-based CeO_2_ nanocomposites are shown in Fig. 1. The Rietveld fitted XRD spectrum of pure CeO_2_ powder sample is well represented in Fig. 1a. The refined results show that this sample has a cubic structure and is also matched with standard JCPDS (ICDD) card number 34-0394. The characteristic peaks of pure CeO_2_ and GO-based CeO_2_ nanocomposites shown in the XRD pattern represent their polycrystalline nature. The diffraction peaks of pure CeO_2_ and GO-based CeO_2_ nanocomposites at angle 2θ = 28.5°, 33°, 47.5°, 56.3°, 59.1°, 69.4°, 76.7° and 79.1° correspond to (111), (200), (220), (311), (222), (400), (331) and (420) Miller planes^44^, respectively (see Fig. 1b). There is a diffraction peak of GO at angle 2θ = 10° attributed to the (001) plane. GO-based CeO_2_ nanocomposite was also confirmed by the presence of both GO and CeO_2_ peaks in the final composite due to the exfoliation of GO^45^.

In the XRD patterns of each sample, no extra peak was observed; also, no detectable shift of the XRD peak was obtained, which indicates that the nanocomposite was formed without any impurity. The lattice parameter (a = b = c), volume size (a^3^), dislocation density (δ = 1/D^2^), and lattice strain ($\varepsilon =\frac{\beta cos\theta }{4}$) were also evaluated and listed in Table 1. The ionic radii of Ce^3+^ and Ce^2+^ are different, so the nonlinear trends of lattice parameters have been observed in the case of all samples, as both the oxidation states of Ce can be confirmed from XPS (see Fig. 7 and 8). The lattice constant for each sample was calculated by using the following (Eq.1),

1 $${d}_{hkl}=\frac{a}{\sqrt{{h}^{2}+{k}^{2}+{l}^{2}}},$$

where the interplanar spacing (d_hkl_) can be evaluated by using Bragg’s law, as expressed below (Eq. 2) and (hkl) are the miller indices of the diffraction planes.

**Table 1:** Lattice parameter, unit cell volume, dislocation density, strain, average crystallite size, and energy band gap for all synthesized samples.

Samples	Lattice parameters (a = b = c) (Å)	Volume size (Å^3^)	Dislocation density (lines/nm^2^)	Strain	Average crystallite size (nm)	Band gap (eV)
CeO_2_	5.400	157.464	0.0025	0.00131	20	2.80
GO-1/CeO_2_	5.403	157.695	0.0021	0.00138	22	2.16
GO-5/CeO_2_	5.420	159.244	0.0019	0.00141	23	1.97
GO-10/CeO_2_	5.414	158.725	0.0017	0.00123	24	1.73
GO-15/CeO_2_	5.417	158.942	0.0016	0.00135	25	1.68

2 $$2{d}_{hkl}Sin\theta =\lambda .$$

The average crystallite size of each sample was calculated using the Debye-Scherer formula (Eq. 3)^46^,

3 $$D=\frac{0.9\lambda }{\beta cos\theta },$$

where *λ* is the wavelength of the X-ray source utilized (1.541 Å), *β* is the full-width-at-half-maximum (FWHM) of the diffraction peak, *D* is the average crystallite size, and θ is the glancing angle. The value of *D* is found to increase from 20 to 25 nm with increasing concentration of the GO (see Table 1). The interaction between graphene oxide and cerium oxide under this synthesis condition could promote the growth of cerium oxide particles. Because utilized synthesis conditions of the graphene oxide lead to agglomeration or clustering of particles, it might give the appearance of larger particle sizes^47^. The increased crystallite size enhances the overall surface area and catalytic activity of the nanocomposites, making them more effective in various applications, such as catalysis and antibacterial.

### Fourier transform infrared (FTIR) spectroscopy

**Figure 2:**
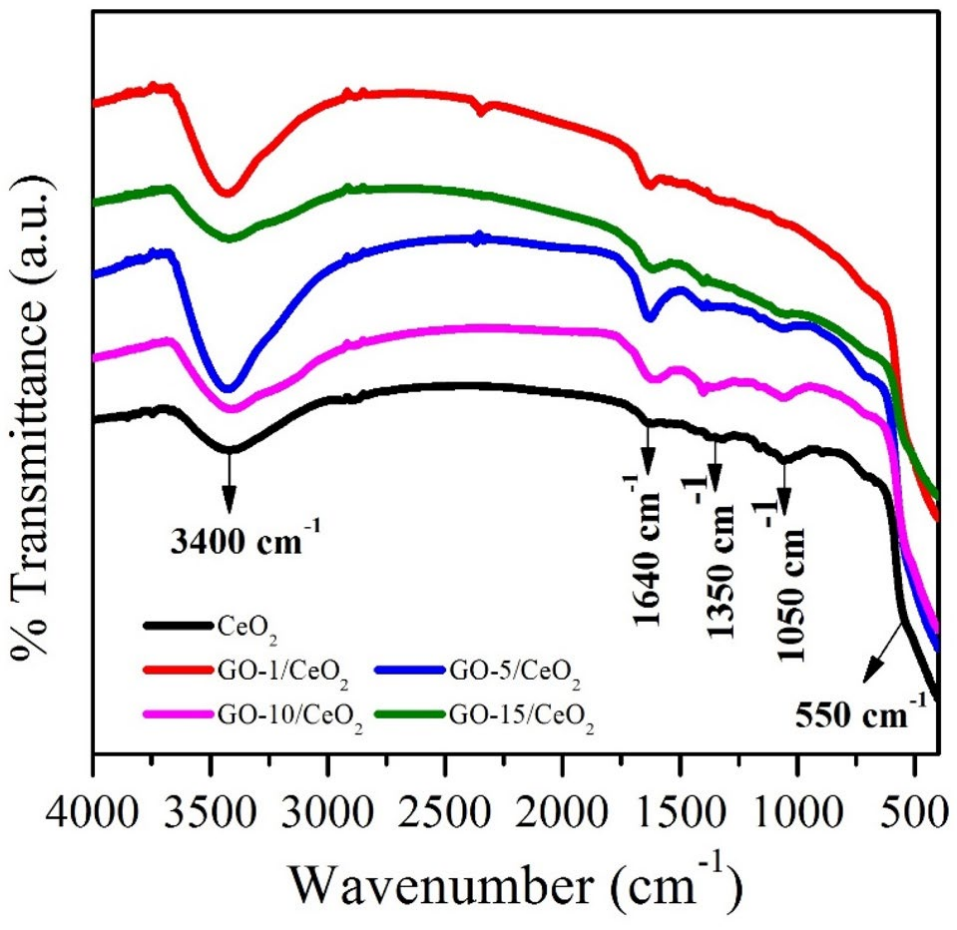
FTIR spectra of pure CeO_2_ NPs and GO (1, 5, 10 and 15 wt.%) based nanocomposite.

The FTIR technique is used to collect data on chemical bonds, vibrational frequencies, and the presence of functional groups and assists in figuring out the material's phase structure^48^. The FTIR spectra of each sample of synthesized materials using the KBr pellet method are shown in Fig. 2. The consistency among the spectra suggests that the CeO_2_ has been successfully decorated on the GO nanosheets. The inter-atomic vibrations in oxide compounds are accounted for the absorption bands below 1000 cm^–1^, which are also known as the fingerprint region of the FTIR spectra^49^. The weak Ce–O vibration in CeO_2_ was indicated by the absorption peak labeled at 550 cm^–1^^50^. Additional peaks at 1350 cm^–1^ and 1640 cm^–1^ correspond to carboxylic acid's weak C–H bending vibrations and intense C=O stretching. The broad peak at 3400 cm^–1^ is associated with the intense O–H stretching vibration of hydroxyls from absorbed water molecules^51^. The functional groups for all GO based CeO_2_ are shown in Table 2.

**Table 2:** Functional group for all synthesized materials.

Synthesized sample	Bands (cm^–1^)			
	Ce–O–Ce	C–H	C=O	O–H
CeO_2_	550	1350	1640	3400
GO-1/CeO_2_	548	1355	1635	3421`
GO-5/CeO_2_	547	1360	1630	3423
GO-10/CeO_2_	542	1370	1627	3425
GO-15/CeO_2_	541	1377	1625	3427

### Raman spectroscopy

**Figure 3:**
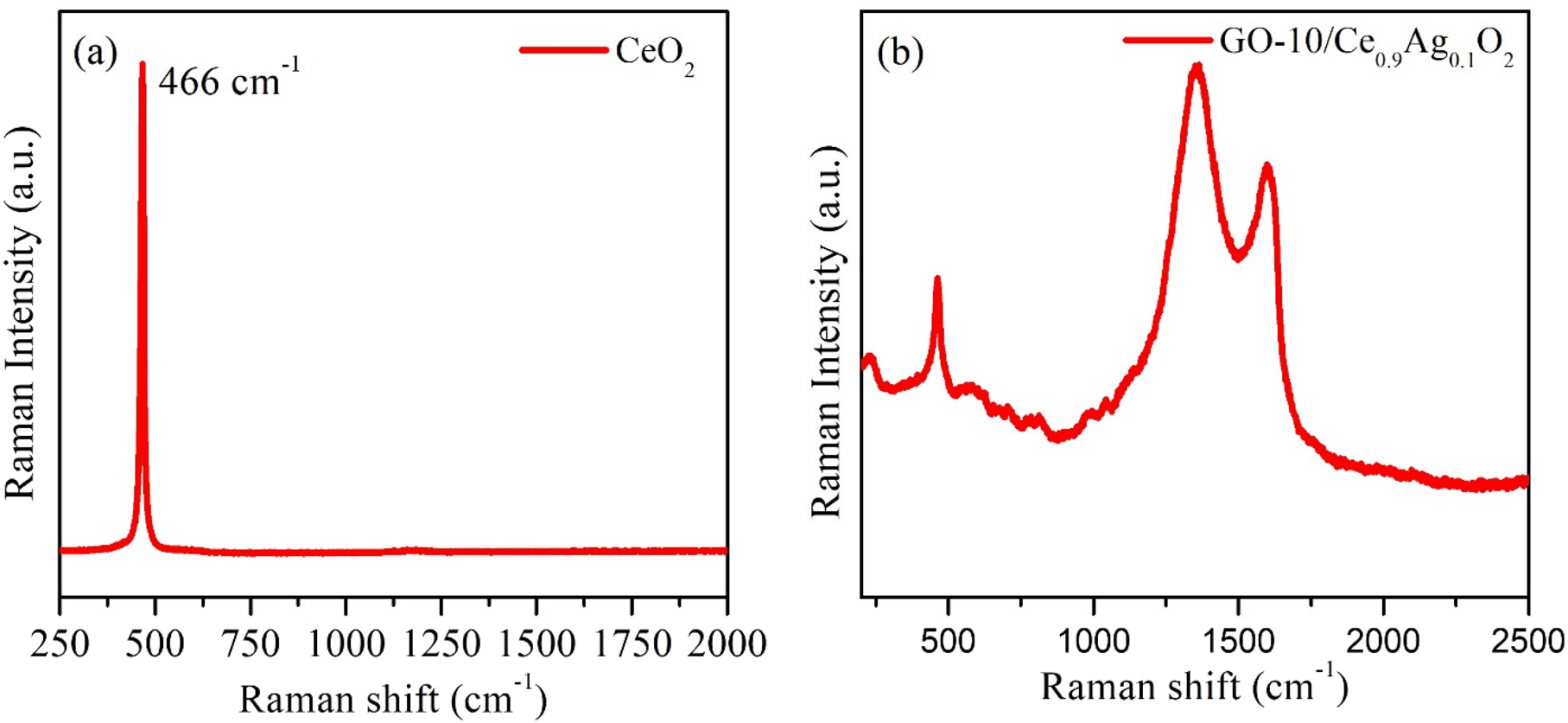
Raman spectra (**a**) for CeO_2_ NPs and (**b**) GO (10 *wt.%*) based CeO_2_ nanocomposite.

Figure 3a,b shows the Raman spectra of CeO_2_ NPs and GO-10/CeO_2_ nanocomposite. In the Raman spectra of CeO_2_ (Fig. 3a), the strong peak at 466 cm^–1^ is associated with the symmetrical stretching mode of the Ce–O vibrational unit^52,53^. The two prominent peaks at 1351.7 cm^–1^ and 1604.7 cm^–1^ are shown in Raman spectra of GO-10/CeO_2_ (Fig. 3b), corresponding to the D and G bands, respectively. The D band is associated with the C sp^2^ atoms’ in-plane vibration^52–55^, whereas the G band is related to structural defects, including bond-angle disorder, hybridization, and bond length disorder that can break the selection principles and symmetry. Defects, such as oxygen vacancies or cerium (Ce) vacancies, create additional states in the bandgap. These states act as trap sites for photoinduced electrons and holes, preventing their recombination and leading to enhanced charge carrier generation, which plays an important role in photocatalytic activity. Also defects in CeO_2_ can lead to the generation of reactive oxygen species (ROS) under light irradiation. ROS, such as superoxide radicals and hydroxyl radicals, have strong antibacterial properties by inducing oxidative stress in bacteria. The blue-shifted peak at 462 cm^–1^ in the GO-based CeO_2_ nanocomposite demonstrates that the CeO_2_ nanoparticles are securely attached to the GO. The charge transfer that takes place between CeO_2_ and GO is responsible for this blue shift^52–54^.

### Transmission electron microscopy (TEM)

**Figure 4:**
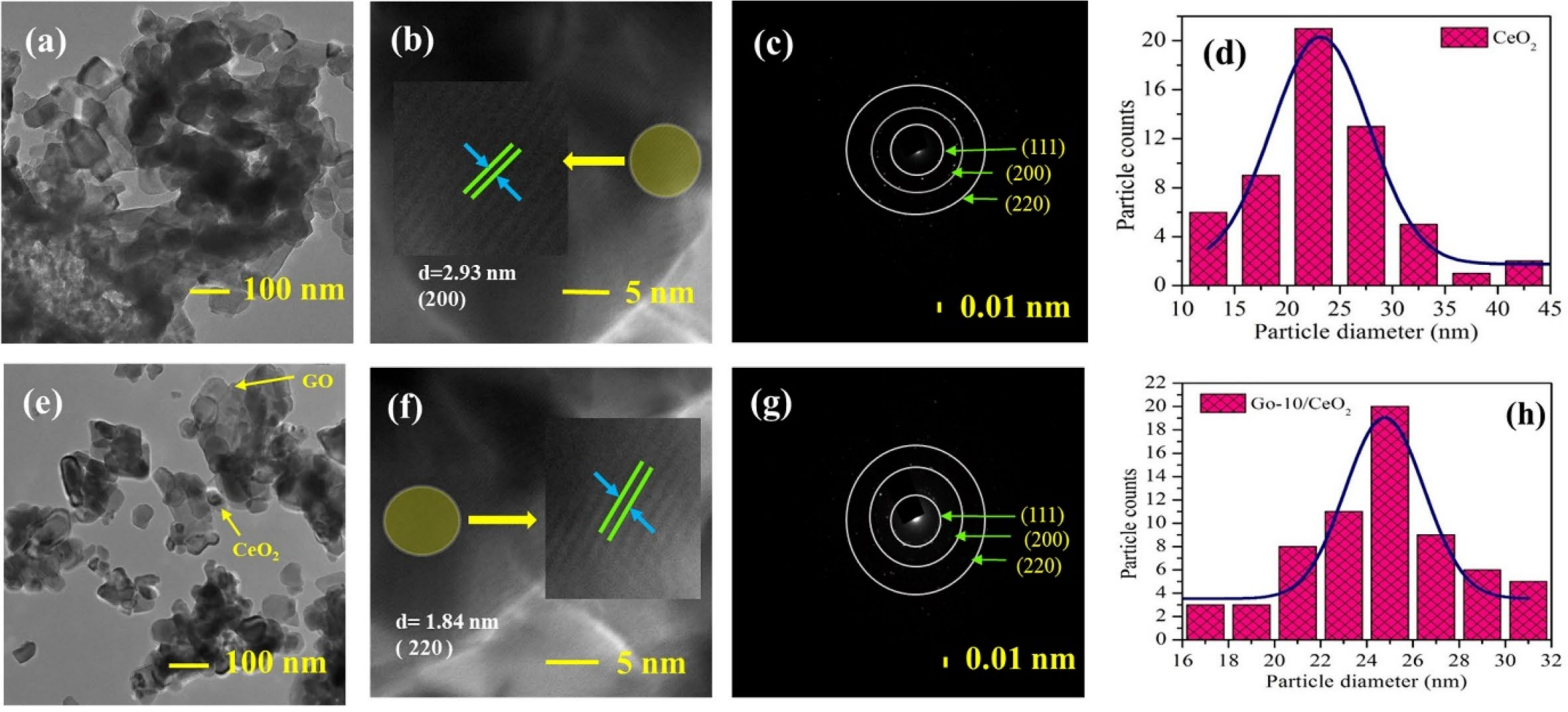
(**a**,**e**) TEM images, (**b**,**f**) HRTEM fringe micrographs, (**c**,**g**) SAED graphs of pure CeO_2_ NPs and GO (10 wt.%) based CeO_2_ nanocomposite, respectively and (**d**,**h**) Gaussian fitting of particle size distribution.

The morphology (particle size & their distribution), lattice fringe patterns, and ring patterns of the CeO_2_ and GO (10 *wt.%*) based CeO_2_ nanocrystalline powder sample were studied using TEM, HRTEM, and SAED techniques, respectively, as shown in Fig. 4. The TEM images shown in Fig. 4a,e corroborated the particle’s spheroidal form and exhibited some distinct aggregation of nanostructured particles. Figure 4b,f analyses the HRTEM fringe patterns at higher magnification (5 nm scale) of CeO_2_ and GO (10 *wt.%*) based CeO_2_, and the insets show the zoomed area of fringe patterns. The measured d-spacings in two directions were 2.93 and 1.84 nm, indicating the (200) and (220) Miller planes of CeO_2_. Figure 4c,g SAED patterns depict ring networks composed of distinct patches, indicating the polycrystalline nanostructure of both pure and GO (10 wt.%)-based CeO_2_. Particle sizes for the sample CeO_2_ and GO (10 *wt.%*) based CeO_2_ were found to be 23 nm and 25 nm, respectively, which is in good agreement with the XRD data, as shown in Fig. 4d,h which displays the curves for the distribution of particle size fitted by Gaussian distribution. According to HRTEM and SAED patterns, all compositions have a cubic bixbyite structure, which is explained by the observed d-spacings and planes^56^.

### Scanning electron microscopy (SEM)

**Figure 5:**
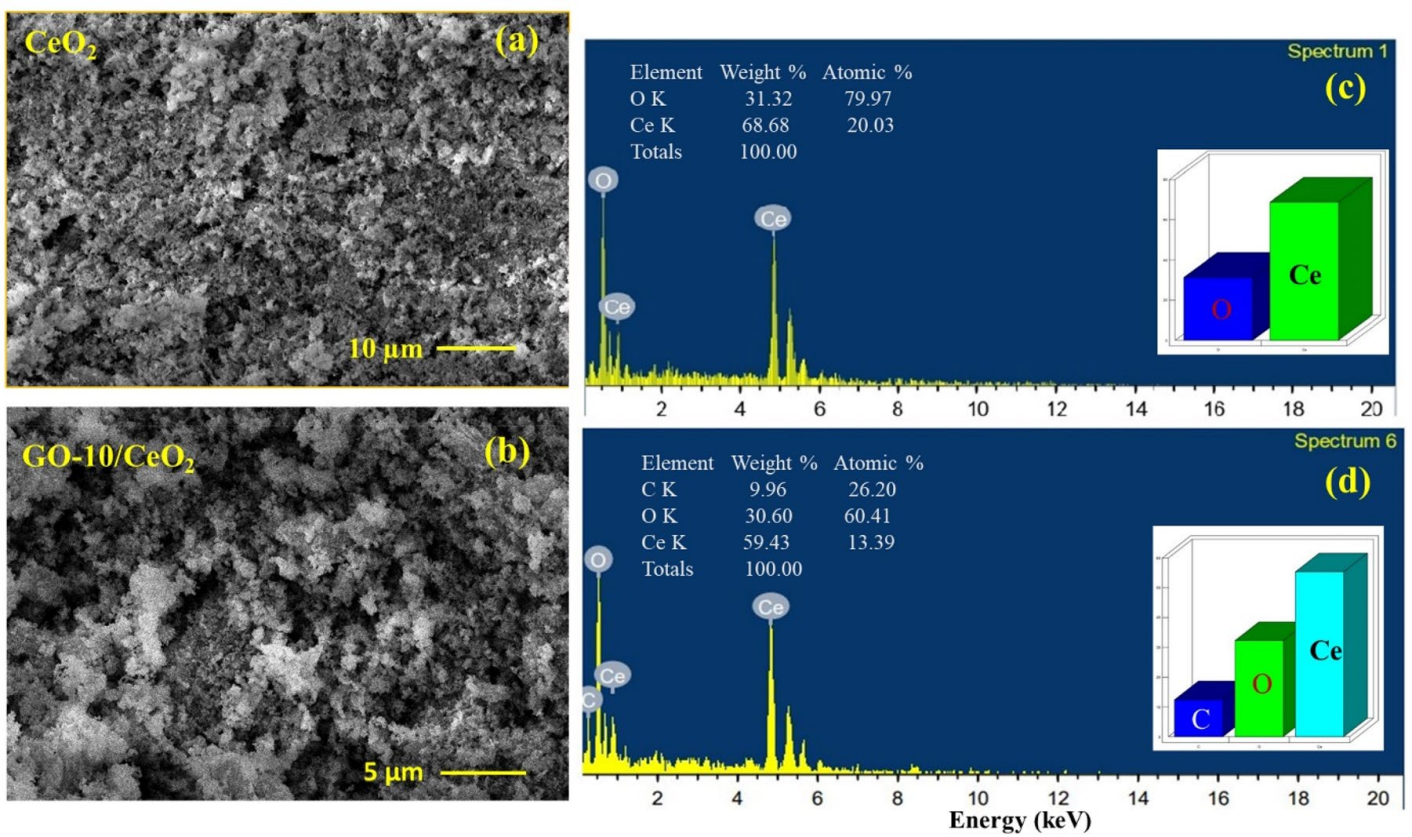
SEM images for (**a**) pure CeO_2_ NPs and (**b**) GO (10 *wt.*%) based CeO_2_ nanocomposite. EDS spectra for (**c**) pure CeO2 NPs and (**d**) GO (10 *wt.*%) based CeO_2_ nanocomposite.

The SEM was used for the morphological analysis of CeO_2_ NPs and GO (10 *wt.%*) based CeO_2_ nanocomposite, as shown in Fig. 5a and b, with the appropriate scale indication at 10 and 5 µm, respectively. The optical, electronic, and structural properties of nanocrystalline materials can be affected by their morphology. In SEM images, the grains appeared to be of different sizes and shapes along with agglomerated nanocrystallites. CeO_2_ showed the agglomeration of synthesized nanoparticles (Fig. 5a). The nanocomposites showed CeO_2_ nanoparticles to be enclosed, attached, and of a porous nature (Fig. 5b). Additionally, it can be seen from the micrographs that the grain sizes get smaller with GO, which is in good agreement with the findings from XRD and TEM. Additionally, the formation of polycrystalline grains from the fusing of several crystallites may account for the variation in crystallite or grain size^57^. It has come to conclude that every sample is uniformly dense, has good crystal quality, and is free of any microscopic defects in the grains.

As shown in Fig. 5c and d, the chemical and elemental composition of CeO_2_ and GO (10 *wt.%*) based CeO_2_ was also examined using EDX connected to SEM. The attached tables in the insets of Fig. 5c and d showed the elemental compositions by weight percent and atomic percent, and the spectra showed that in the synthesized powder samples, the only elements present are Ce, O, and Ce, O, and C for CeO_2_ and GO (10 wt.%) based CeO_2_, respectively^58^.

### UV–visible spectroscopy

**Figure 6:**
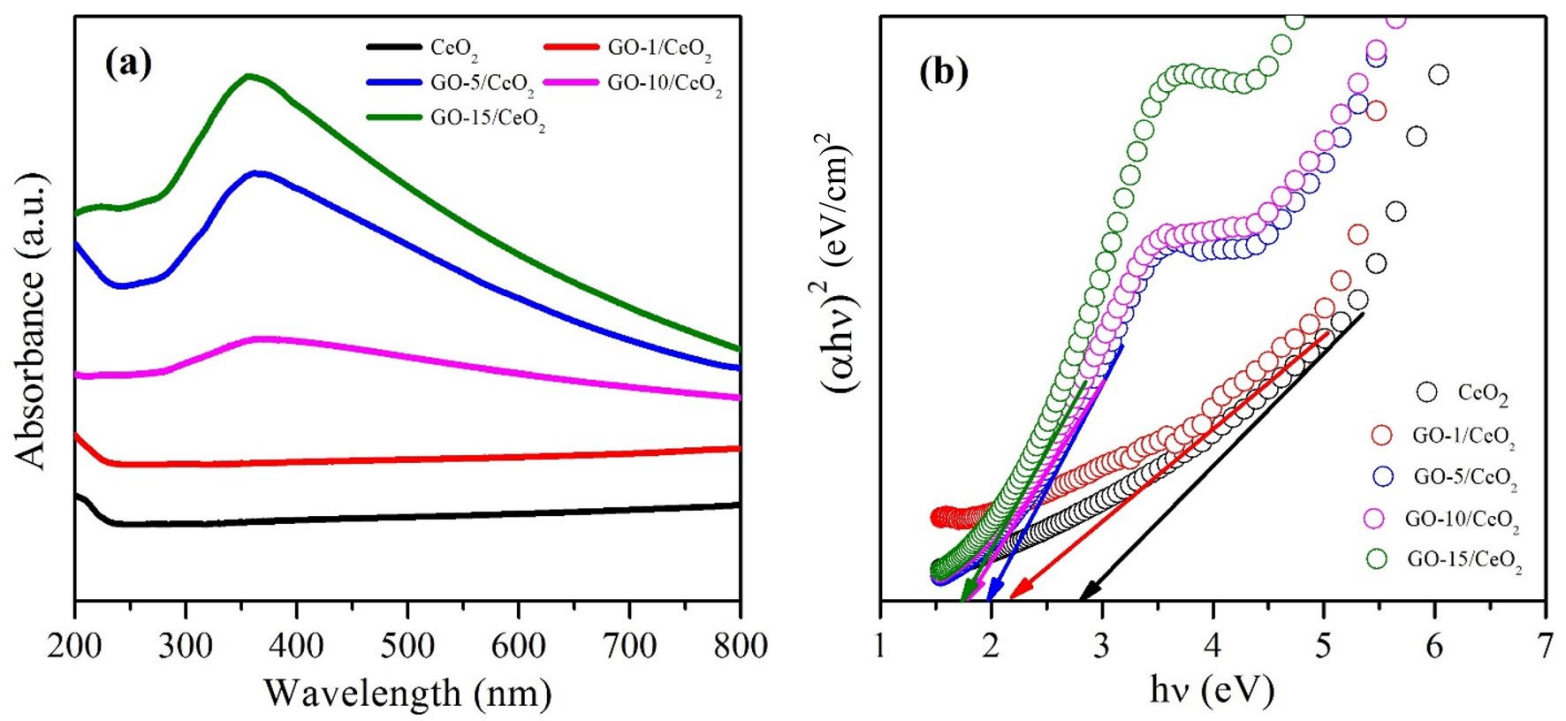
(**a**) UV–vis absorbance spectra of all synthesized materials. (**b**) Tauc plot for energy band gap calculation of each sample.

As shown in Fig. 6, optical absorbance spectra of the synthesized samples were performed using a double-beam UV–Vis spectrophotometer to study the photocatalytic activities and optical properties of these samples. In Fig. 6a, the absorbance at 340 nm is caused by the scattering effect of the randomly organized grain boundaries and nanocrystallites. The average crystallite size was increased with the incorporation of GO nanosheets, which was concomitant with the decreasing behavior of the optical band gap (from 2.8 to 1.68 eV) due to the quantum confinement effect. With the increased GO concentration, a slight red-shift was seen in the absorbance edge, indicating a corresponding contraction of the band gap^59^ as shown in Table 1. In order to be a promising photocatalyst and exhibit effective photocatalytic properties, the material must be optically active (to produce electron–hole pairs). CeO_2_ is a direct band gap semiconductor. So, we utilized this Tauc equation for calculating the band gap for n = 1/2. As shown in Fig. 6b, the optical energy band gap E_g_ of the synthesized sample was calculated using Tauc's plot and the relation (Eq. 4).

4 $$\alpha h\nu =B{(hv-{E}_{g})}^{1/2},$$

where

5 $$\alpha = \frac{2.303 \times A}{t}.$$

Here, *hv, B, E*_*g*_*, α, t*, and *A* are the energy of an absorbed photon in *eV*, energy independent constant, band gap, the coefficient of the absorption (Eq. 5), quartz cuvette thickness (*10 mm*), and sample absorbance, respectively.

### X-ray photoelectron spectroscopy (XPS)

**Figure 7:**
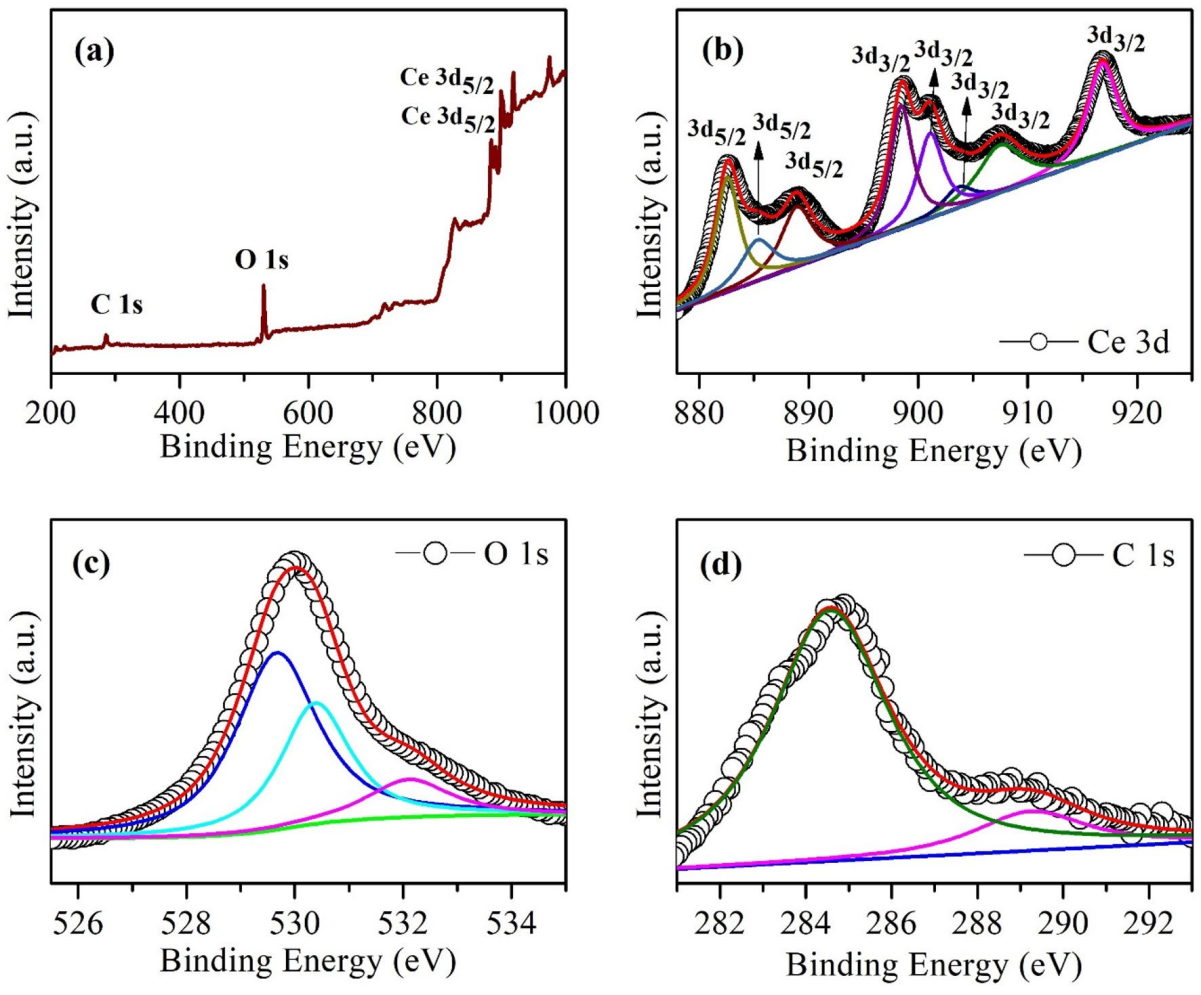
XPS (**a**) full scan spectrum, narrow scan spectra of (**b**) Ce 3d, (**c**) O 1 s and (**d**) C 1 s energy level of CeO_2_ NPs.

**Figure 8:**
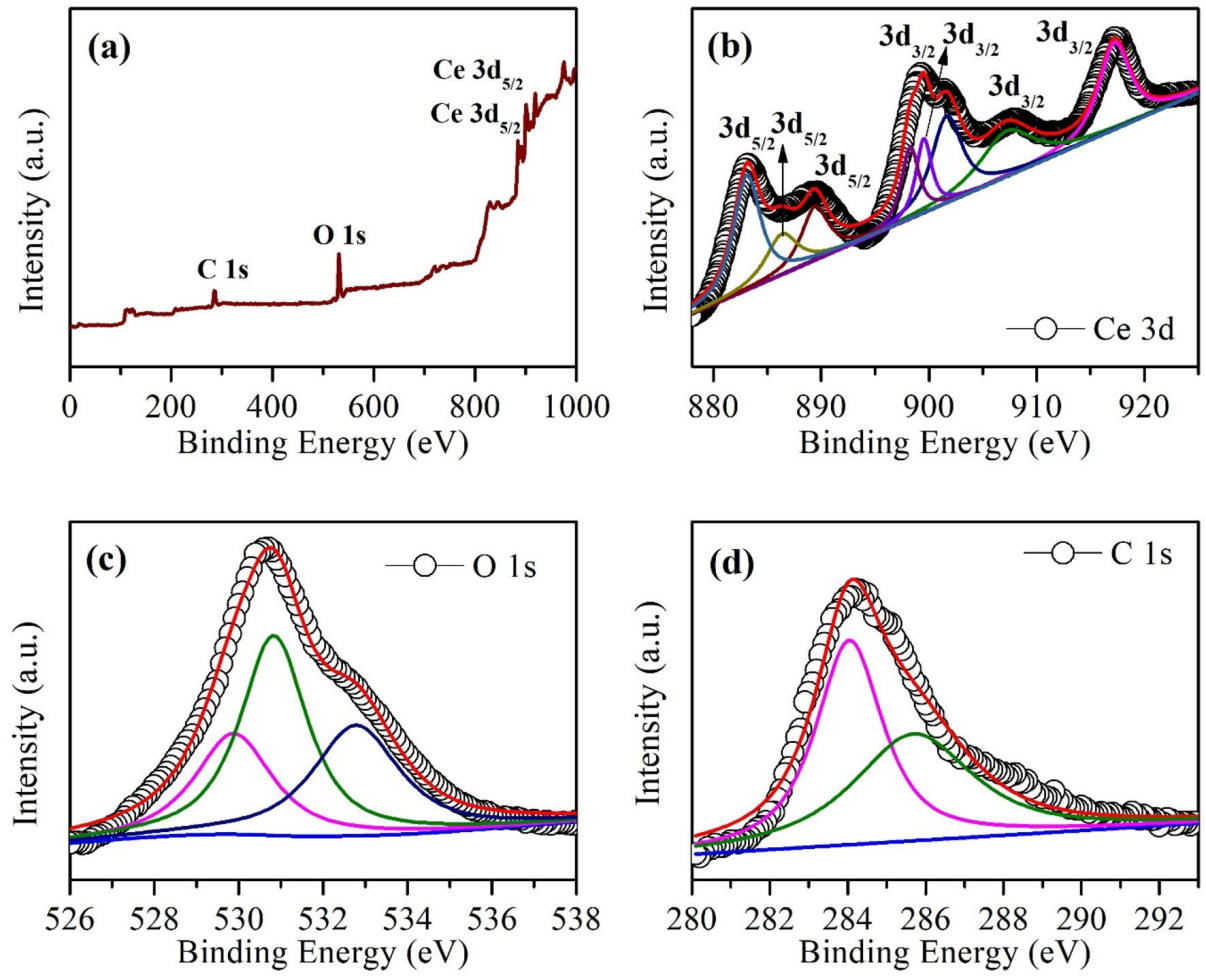
XPS (**a**) full scan spectrum, narrow scan spectra of (**b**) Ce 3d, (**c**) O 1 s and (**d**) C 1 s energy level of GO (10 *wt.*%) based CeO_2_ nanocomposite.

The surface elemental compositions of CeO_2_ and GO-10/CeO_2_ are further studied by using XPS, which confirms that the sample contains cerium, carbon, and oxygen elements without other impurities, as can be seen in Fig. 7, 8. The analysis of the CeO_2_ and GO-10/CeO_2_ nanocomposite spectra showed the presence of Ce 3d, C 1 s, and O 1 s in Fig. 7 and 8, respectively. The spin–orbit splitting of Ce 3d_5/2_ and Ce 3d_3/2_^60^ might account for eight peaks in the high-resolution spectra of Ce 3d (Fig. 7b and 8b). Consequently, CeO_2_ and its nanocomposite include both oxidation states Ce^3+^ and Ce^4+^ due to spin doublet splitting^61^. (882.6, 885.3, 888.9, and 898.5 eV) for CeO_2_ and (883.1, 886.3, 889.4, and 898.3 eV) for GO-10/CeO_2_ nanocomposite correspond to the Ce 3d_5/2_ ionization, whereas (901.1, 903.7, 907.5, and 916.8 eV) for CeO_2_ and (899.5, 901.6, 907.2 and 917.2 eV) for GO-10/CeO_2_ nanocomposite correspond to the Ce 3d_3/2_ ionization. Ce^4+^ 3d states are shown by the peaks with binding energy (BE) values of 882.6, 888.9, 898.5, 901.1, 907.5, and 916.8 eV for CeO_2_ and 883.1, 889.4, 898.3, 899.5, 907.2, and 917.2 eV for GO-10/CeO_2_ nanocomposite, showing that + 4 is the predominant valence state of Ce in the sample^32^. At (885.3 and 903.7 eV) and (886.3 and 901.6 eV) for CeO_2_ and its nanocomposite, respectively, the XPS peaks correspond to the valence state of Ce^3+^^62^. Both Ce^3+^ and Ce^4+^ oxidation states can play distinct roles in photocatalytic phenomena. Ce^3+^ is often involved in redox mediation and electron scavenging, while Ce^4+^ can act as an electron acceptor, contributing to the overall efficiency of photocatalytic processes. The specific benefits depend on the photocatalytic system and the reaction involved.

The intense peaks at 529.6 and 529.8 eV, in the high-resolution O 1 s XPS (Fig. 7c, 8c), are related to lattice O of CeO_2_ and GO-10/CeO_2_, respectively^63^; however, the high BE (530.4 eV and 532.1 eV) and (530.8 And 532.8 eV) peaks may be due to hydroxyl or water or loosely bound adsorbed oxygen present in the unoccupied sites of the lattice and also due to oxygen deficiency region inside CeO_2_ matrix (O attached to Ce^3+^). Two peaks with BE values of (284.6 and 289.2 eV) and (284.0 and 285.7 eV) were fitted to CeO_2_ and GO-10/CeO_2_ C 1 s spectra, corresponding to sp^2^ bonded carbon C–C and N–C=N bonds, respectively (see Fig. 7d, 8d)^64^.

### Photocatalytic activity

Wastewater treatment by using the photocatalytic technique is described as follows: About 20 mg of photocatalysts, CeO_2_, and its GO (1, 5, 10, and 15 *wt.%*) based nanocomposites were mixed throughout a solution of 1000 mL distilled water containing 10 mg of methylene blue (MB) dye. The pH of the solution was set to be around 7 after degradation. This solution was vigorously stirred for 30 min in the dark to attain a state of equilibrium between the photocatalyst and dye. Sunlight was thrown into this solution while it was being stirred after the adsorption and desorption processes had reached equilibrium. A Small amount of the solution was taken after a predetermined period of time. Centrifugation was employed to separate the photocatalyst from the treated solution, and UV–vis measurements of dye degradation were recorded using a spectrophotometer. The procedure was repeated to complete the degradation of MB dye, and no further absorption peak could be detected.

**Figure 9:**
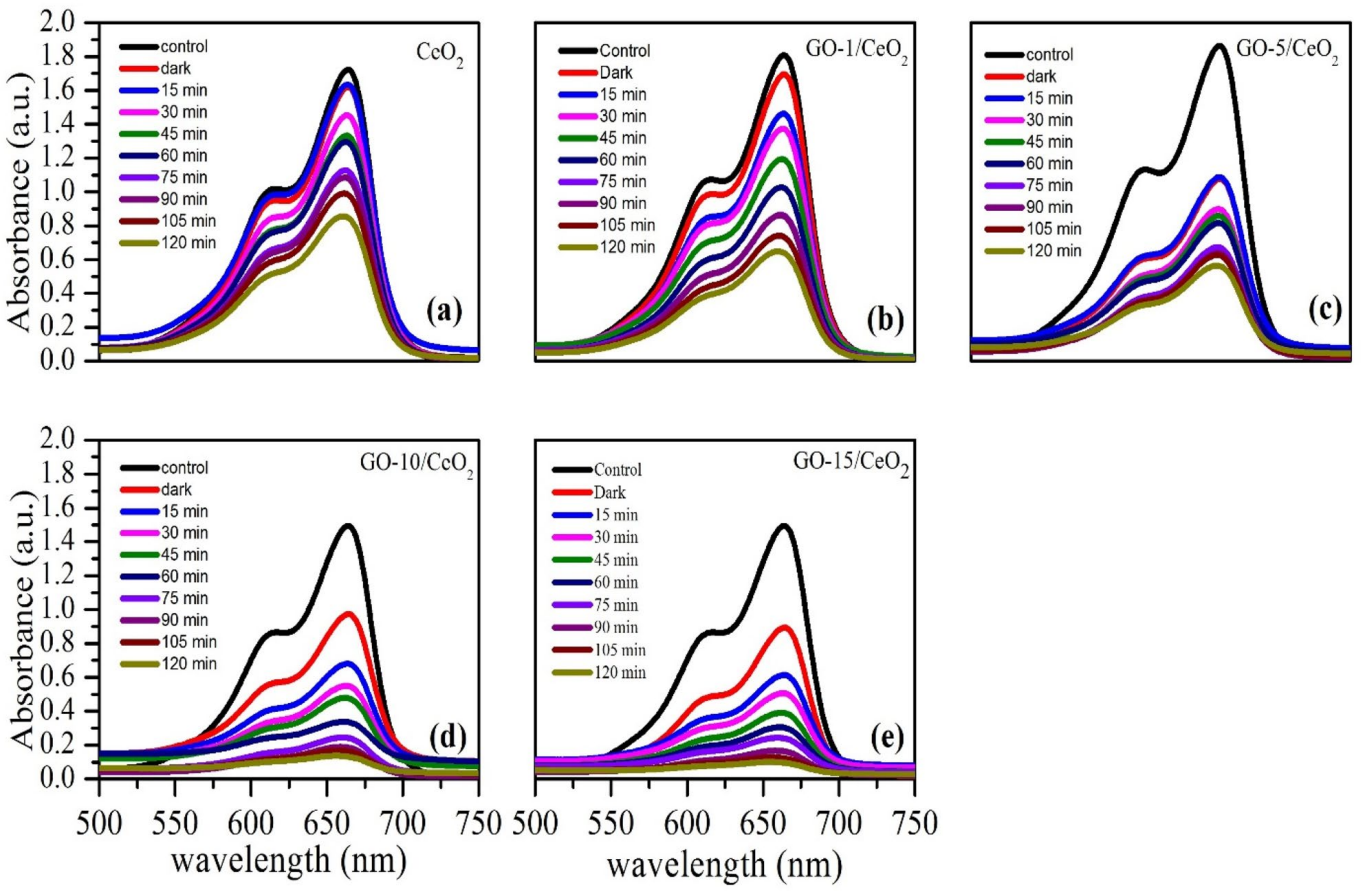
Absorbance spectra of MB dye after wastewater treatment in the presence of sunlight with photocatalyst (**a**) pure CeO_2_ (**b**) GO based (1 *wt.*%) CeO_2_ (**c**) GO based (5 *wt.*%) CeO_2_ (**d**) GO based (10 *wt.*%) CeO_2_ and (**e**) GO based (15 *wt.*%) CeO_2_, respectively.

**Figure 10:**
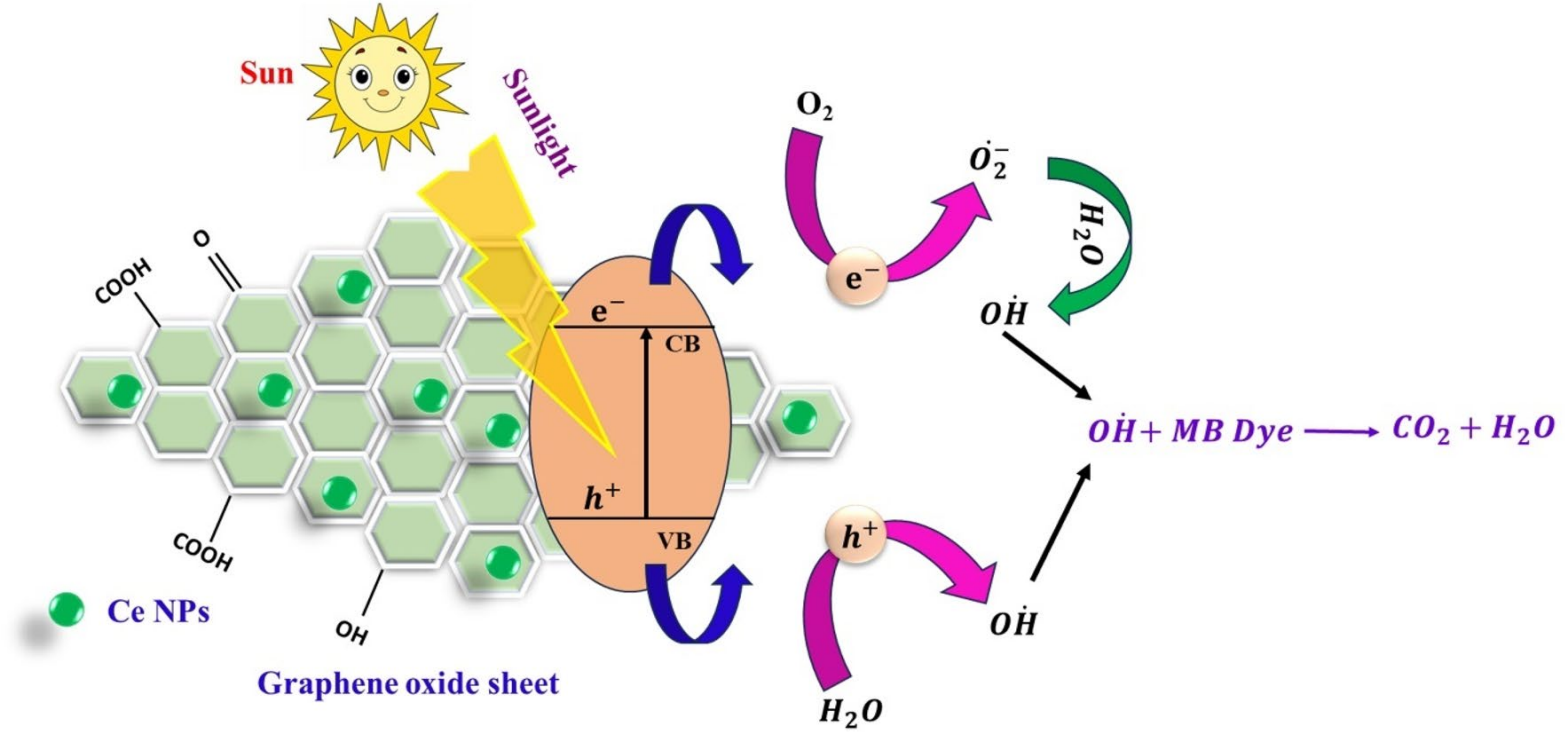
Mechanism of photodegradation of MB dye using synthesized materials.

Figure 9a–e shows the UV–Vis absorption spectra with photocatalysts CeO_2_ and GO-based CeO_2_ nanocomposites for MB dye degradation after exposure to sunlight for different time intervals. The absorption peaks at 663 nm^65^ for MB dye degradation show that as the exposure duration of sunlight to solution increases, the intensity of peaks decreases, indicating the degradation of MB dye in the presence of sunlight. When the sun radiation with a proper amount of energy (comparable to or greater than the energy of the band gap) reaches the surface of the photocatalyst, electron–hole pairs are produced, which are required to start the photocatalytic process. The excited electrons in the conduction band and holes in the valence band react with nearby oxygen (O_2_) molecules and surface-bound water molecules to create superoxide radical anion ($\dot{{O}_{2}^{-}}$) and hydroxyl radical ($\dot{OH}$). Free radicals shown in equation (Eq. 6) are referred to as ROS (reactive oxygen species)^66^. They react quickly with organic pollutants to break them apart^67^, and Fig. 10 shows the mechanism of photocatalytic activity.

6 $$\dot{OH}+MB\left(dye\right)\to {H}_{2}O+C{O}_{2}.$$

It is possible to use this photocatalyst to degrade all dyes, such as methyl red, rhodamine B, congo red, methyl orange, methyl violet, acid Blue 80, malachite green, etc., that are susceptible to degradation in the presence of these reactive radicals^68^.

**Figure 11:**
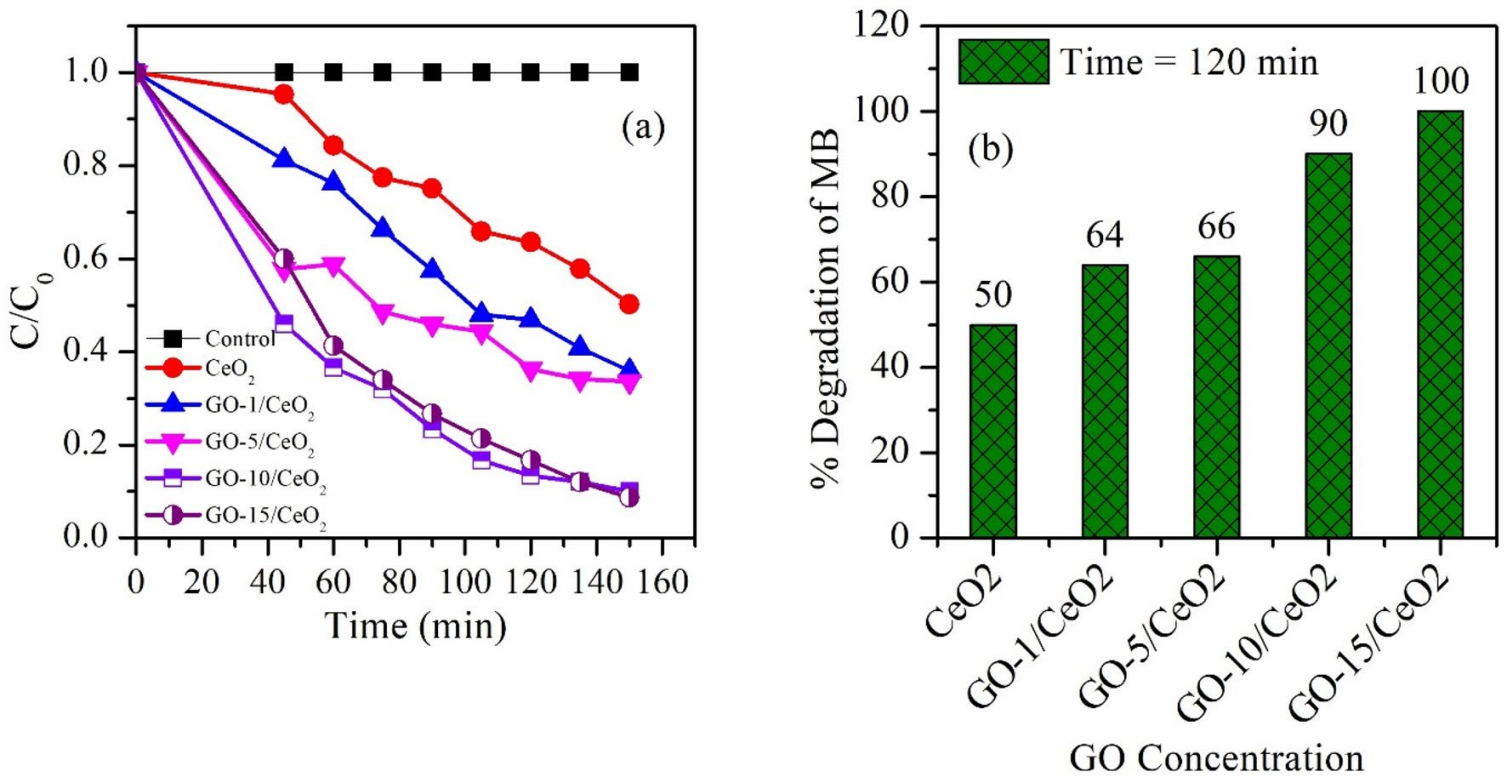
(**a**) C/C_o_ vs time spectra for pure CeO_2_ NPs and GO (1, 5, 10 and 15 *wt.*%) based CeO_2_, and (**b**) Bar diagram of percent degradation vs GO concentration for all synthesized materials.

The photocatalyst can be separated from the solution by centrifugation once the MB dye has been entirely degraded. Figure 11a shows how the "degradation efficiency" (C/C_o_) of the MB dye with time. The increased photocatalytic activity with increasing GO concentration may be due to the following reasons: (i) due to the emergence of generalized localized states, photon absorption increases, and (ii) higher concentrations of oxygen vacancy, which serve as energy traps and slow down recombination of electron–hole pair^52^.

Oxygen vacancies reduced electron–hole recombination and enhanced the photocatalytic activity of CeO_2_ NPs and GO-based CeO_2_ nanocomposites^69^. As shown in Fig. 11b, a pure CeO_2_ photocatalyst degrades the MB dye up to 50%, while GO (1, 5, 10, and 15 wt%) based CeO_2_ photocatalysts degrade 64%, 66%, 90%, and 100% (Table 3), respectively. Here it can be noted that 15% GO-based CeO_2_ nanocomposite is the best photocatalyst to treat the MB dye present in wastewater.

The following expression (Eq. 7) was used to determine the proportion of MB dye that was degraded due to the presence of photocatalysts,

7 $$\% Degradation=\frac{{C}_{0}-{C}_{t}}{{C}_{0}}\times 100,$$

where ${C}_{0}$ and ${C}_{t}$ represent MB dye concentration at 0 min and 120 min, respectively, before and after degradation. The MB dye photocatalytic degradation reaction rate constant, k, was calculated using pseudo-first-order rate kinetics. The pseudo-first-order kinetic model is commonly used to describe the kinetics of photocatalytic reactions. In the context of photocatalytic activity, this model is often applied to describe the degradation or transformation of a target compound (e.g. MB dye) under the influence of a photocatalyst (e.g. CeO_2_ and GO-based CeO_2_ nanocomposites). The natural logarithmic transformation of the concentration ratio (${\text{ln}}\left(\frac{{C}_{0}}{{C}_{t}}\right)$) on the y-axis results in a linear relationship with time (*t*) on x-axis. This linear fitting allows for easier analysis and determination of the pseudo-first-order rate constant, i.e., equal to the value of the observed slope. It can be explained by the following expression (Eqs. 8 and 9),

8 $${C}_{t}={C}_{o}{e}^{-kt},$$

9 $${\text{ln}}\left(\frac{{C}_{0}}{{C}_{t}}\right)=kt.$$

**Figure 12:**
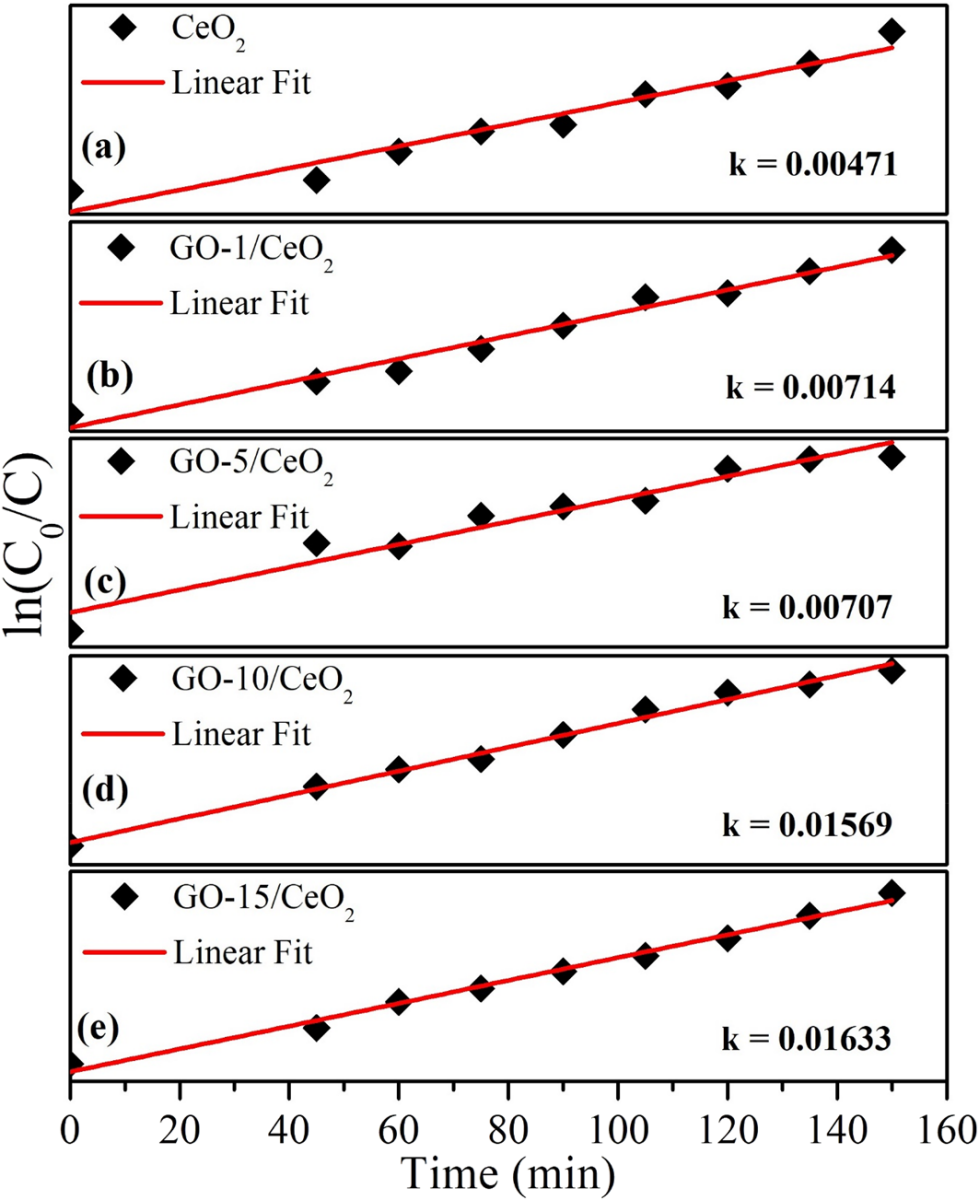
Reaction rate constant for the photocatalysts (**a**) pure CeO_2_ (**b**) GO (1 *wt.*%) based CeO_2_ (**c**) GO (5 *wt.*%) based CeO_2_ (**d**) GO (10 *wt.*%) based CeO_2_ and (**e**) GO (15 *wt.*%) based CeO_2._

As seen in Fig. 12a–e, the rate constants for CeO_2_ and GO (1, 5, 10, and 15% wt.%) based CeO_2_ were found to be 0.00471, 0.00714, 0.00707, 0.01569, and 0.01633 min^–1^, respectively. Faster photodegradation of MB dye is attributed to the presence of GO (15 wt.%) because of the suppression of the electron–hole pair recombination rate caused by the development of localized energy states and oxygen vacancies^70^.

**Table 3:** Degradation of MB dye using different photocatalysts and antibacterial activity obtained from previous studies.

Photocatalyst	Degradation efficiency (%)	Reaction time (min)	Initial MB dye concentration (mg/L)	Refs.	ZOI (mm)	Refs.
ZnO NPs	96.5	100	15	^71^	12 for *E. coli*	^72^
CuO NPs	61	120	10	^73^	14.3 ± 0.5 for *S. aureus*	^74^
CeO_2_ NPs	77	210	12.5	^75^	3.33 ± 0.33 for *P. aeruginosa*	^76^
N-ZrO_2_ NPs	59	240	5	^77^	–	–
GO-15/CeO_2_	100	120	20	Present work	11.24 ± 0.14	Present work

### Antibacterial activity

#### Determination of zone of inhibition

**Figure 13:**
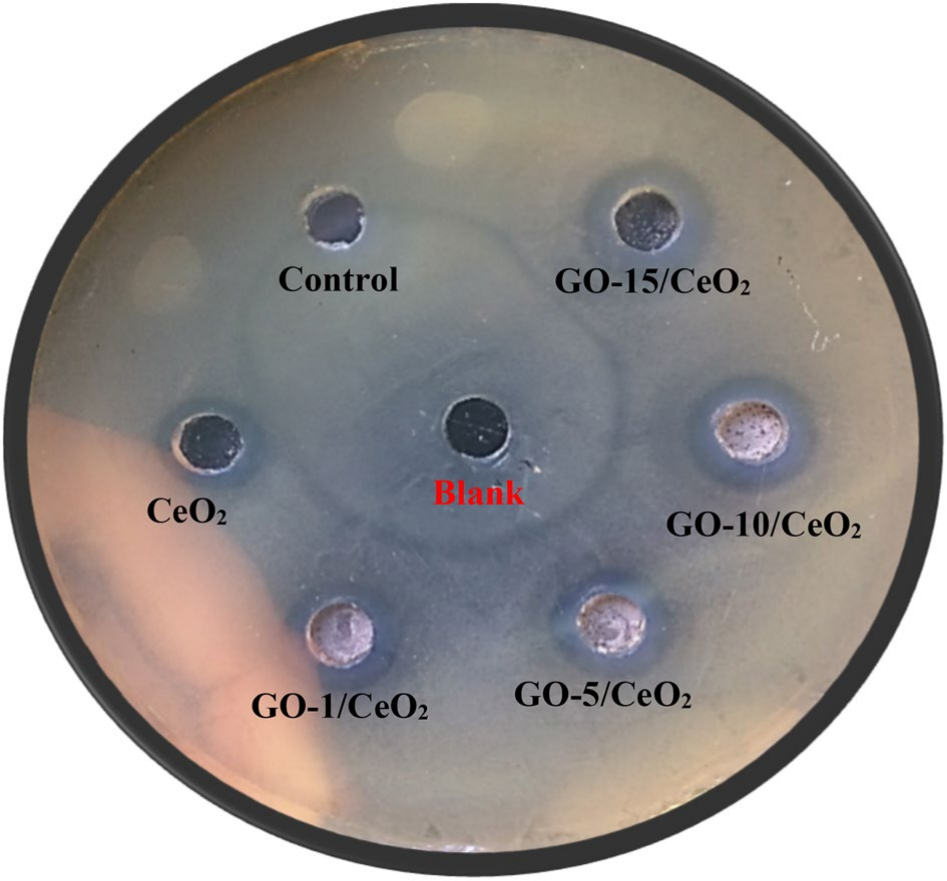
Antibacterial activity of nanoparticles against *Pseudomonas aeruginosa* PAO1.

Nanoparticles were evaluated for antibacterial efficacy against pathogenic bacteria PAO1 using the well-diffusion method. Table 4 shows the results of the antibacterial activity evaluation of nanoparticles. According to the findings, some nanoparticles were potentially beneficial in reducing bacterial development (Fig. 13).

**Table 4:** Nanoparticles with their zone of inhibition (mm) and MIC (µg/mL) value against the pathogenic bacteria.

S. no	Nanoparticle	Antibacterial activity against PAO1	
		Zone of inhibition (mm) 50 (μg/mL)	MIC (µg/mL)
1	Control	0.00	–
2	Blank	0.00	–
3	CeO_2_	8.47 ± 0.15	20
4	GO-1/CeO_2_	9.61 ± 0.12	20
5	GO-5/CeO_2_	10.35 ± 0.11	15
6	GO-10/CeO_2_	10.89 ± 0.10	15
7	GO-15/CeO_2_	11.24 ± 0.14	10

(–) not determined.

#### Determination of MIC

The antibacterial actions of nanoparticles on bacteria are summarized in Table 4. The microbiological sensitivity to the various nanoparticles shown by the mean MIC values ranged from 10 to 20 µg/mL.

## Conclusion

In conclusion, the high-performance photocatalysts CeO_2_ NPs and GO (1, 5, 10, and 15 wt.%) based CeO_2_ nanocomposite were successfully synthesized. The HRTEM, Rietveld refined XRD, Raman spectra, and SAED were used to confirm the single-phase cubic structure of the synthesized materials, and no possible impurities were identified. The average crystallite size was increased with the incorporation of GO nanosheets, which was concomitant with the decreasing behavior of the optical band gap (from 2.8 to 1.68 eV) due to the quantum confinement effect. The FT-IR and Raman analysis confirmed the presence of Ce–O bond and other vibrational modes in each sample. The aggregation of nanoparticles with some porosity in GO-based CeO_2_ nanocomposites was measured in the SEM micrographs. Ce^3+^/Ce^4+^ valence states and the presence of oxygen vacancies were confirmed using XPS measurement. Furthermore, enhanced photocatalytic and antibacterial activities were observed due to the increasing concentration of GO in the pure CeO_2_ NPs because of the formation of oxygen vacancies and localized energy states that slow the recombination rate of electron–hole pairs. The highest rate constant (*k* = 0.01633 min^–1^) was calculated for GO-15/CeO_2_, which degraded the MB dye from wastewater up to 100% in 120 min under sunlight irradiation. Again, these nanocomposites also showed excellent antibacterial activity against *Pseudomonas aeruginosa* PAO1. In particular, these materials could be used in the development of new antibacterial coatings for medical devices, implants, and wound dressings, along with industrial wastewater treatment.

## Data Availability

The datasets generated during and/or analyzed during the current study are available from the corresponding author on reasonable request.
